# 

**Published:** 1891-04-25

**Authors:** 


					43 THU HOSPITAL. April 25, 1891.
NOTICE TO CORRESPONDENTS.
All MS., Letters, Books for Review, and other matters intended for the
Editor should be addressed The Editor, Tee Lodge, Poechestee
Squabe,London, W.
The Editor cannot undertake to return rejected M.S., even when
accompanied by stamped directed envelope.
Notice to Advertisers and Subscribers.
All Advertisements, Orders for Copies of The Hospital, and Business
Communications should be addressed to The Publisher, not to the Editor,
Thf Hohpitai, 140, Strand, W.O.
The Cost of Subscription, post free, is as followsPer week, lid.;
fer quarter, Is. 8d. ; per half-year, 3s. 3d.; per annum, 6s. 6d.
oreipn Per annum, 8s. 8d.; payable, in all cases, in advance.
NOTICE.-The Index for Vol. IX.. fending1 March, 1891)
is on sale at the Office of this Journal, price 2^d.,
post free.
General Charities.
rThia flnln-mn is devoted to an account of work of charitable institu-
tions other than Medical Charities. The Editor will be glad to receive
re^rts or news of work at 140, Strand. Philanthropy should he
plainly written on the envelope.]
The Earl of Lathom will preside at the dinner of the KOYAXi
SOCIETY OP MUSICIANS OP GREAT BEITAIJST, at the
Whitehall Rooms of the Hotel Metropole, on Wednesday, the 29th
inst.
A Flower Ball is to he held on Tuesday, May 5th, in the "Whitehall
Rooms of the Hotel Metropole, for the benefit of the HOME OP
EEST POR HOESES, which is situated at Friars Place Farm,
Acton. The chief object of the institution, which was established five
years ago, is to procure rest, and, when necesEary, veterinary treatment,
for horses which are the property of the poorer classes, so as to enable
them to recover their strength and health, and be of service to their
masters. Before the Home was established no attempt was made to
alleviate the sufferings of such horses as were considered to be worn
out ard fit only for the knacker's yard, whereas, since the Home has
stepped in, many animals have been restored to health, so as to be of
great service to their owners. Contributions will bo thankfully received
y the Secretary, 185, Victoria Street, S.W.
Scraps and Gleanings.
The influenza is spreading: rapidly in Yorkshire and Lincolnshire.
A successful hall was given in Sonthse i, on the 2nd inst., in aid of
the funds of St. John's Convalescent Home.
Professor Corfield has been electee! an honorary member of the
Imperial Society of Medicine of Constantinople.
At the festival dinner of the North London Consumption Hospital
donations amounting to upwards of ?2,500 were announced.
The Dnchess of Teok has consented to become patroness of St.
Monica's Home Hospital for Ch ildren, Brondesbury Park, Kilburn.
On the play-bills of several London theatres there now appears the
announcement that the house is disinfected by the Sanitas Company.
The National Vigilance Association opened a Medical Home for
Women at 179, King's Road, Camden Road, N.W., on Tuesday, April 14th.
The Queen will visit Derby on Whitsnn Thursday, to lay the founda-
tion stone of the new Infirmary. The ceremony will take place about
six p.m.
The old Etonians in Manchester have placed a cot in the children's
ward of Ancoats Hospital, to be supported by them and known as the
Eton Cot.
The London Vegetarian Society defended the principles it advocates
at a publio debate, held in the Memorial Hall, Farringdon Street, on
Thursday last.
The returns of the Registrar-General for the week ending April 11th,
state thit 2,208 births and 1,723 deaths were registered in London
during the week.
A meeting in connection with the Geneva Medical College, was held in
Reading on the 20th, when an Armenian lady and gentleman were
among the speakers.
Sir Bernhard Samuelson, who was Chairman of the Royal Commis-
sion on Technical Education, has offered ?3,000 towards establishing
technical schools at Banbury.
The anniversary festival of King's College Hospital was held on the
10th inst. The Lord Mayor, who presided, announced a list of sub-
scriptions and donations amounting to ?2,383.
A lady, whose name has not yet been disclosed, has forwarded ?1,000
to the Chairman of the Portsmouth Eye and Ear Infirmary, to be de-
voted to increasing the accommedation for out-patients.
A sale of work in aid of the Irish Distressed Ladies' Fund will be
held, by permission of the Duke of Devonshire, in Devonshire House,
Piccadilly, on April 28th and 29th. The sale will be opened by Princess
Louise.
Dundee is at present visited by a severe attack of typhoid fever, and
the Police Commissioner of the city has pensioned the widow of a
sanitary official who died from the disease, which he contracted in the
disoharge of his duty.
A three days' fancy bazaar will take place on April 28th, 29th, and
30th, at Blackheath, to pay oil the debt of ?1,000 on the building fund
of the Blackheath and Charlton Cottage Hospital, The bazaar is
expected to be opened by the Duchess of Albany.
In connection with the refusal of Her Majesty's Ministers to appoint
any women en the Labour Commission, it is interesting to note that
colonial experience is against them. On the Royal Commission on
Labour, now sitting at Brisbane, there is a Lady Commissioner.
The twentieth annual report of the Mablethorpe Convalescent Home
states that the Committee have recei ed from Canon Pretyman the
valuable gift of a closely adjacent cottage, which can, at a small expense,
be fitted up as an isolation ward in case of any patient developing an
infeotious disease.
The Jacksonian Prize of the Royal College of Surgeons has been
awarded to Mr. William Thorburn, F.R.C.S., assistant surgeon to the
Manchester Royal Infirmary. This is only the second time since its
foundation that thi* important prize has been awarded to a Manchester
surgeon, and the first time it has been gained by a graduate of the Man-
chester Medical School.
Dr. Wright, of Glasgow, reports that in a tenement in Pembroke
Street he has 25 patients suffering from irritant poisoning, and he thinks
that the poison has been conveyed through the medium of the milk.
He discovered that the milk came from a farm where there was a cow
recently seized with fever. The milk supply from this souroe was at
once stopped, and the patients are now out of danger.
The eighty-third annual report of the Denbighshire Infirmary states
that the number of in-patients in 1S90 was 147, and the number <? out-
patients 2,180. The total income from ordinary sources was
16s. 8d., and the total expenditure ?1,318 15s. 10d., exceeding the lcceme
by ?177 19s. 2d. This deficiency is met, however, by two legacies oi
?100, and the balance brought forward from last. year.
Colonel Gildea, of Knaresborough Place, S W., Chairman of the
Soldier and Sailor's Families Association, makes an appeal lor funds
to supply the following necessaries urgently wanted for the military
hospitals at Malta, viz., two invalid wbeel-chairs, six screens, a sup-
ply of fly whisks for the summer, six book cases, a supply of suitable
books, warm jerseys, mufflers, and caps, for very sick men, for the
voyage ho me.
Dr. Tatham, of Manchester, in his capacity of Health Officer,deposits
weekly a general health return and " spoi map" at each of the free
libraries and public reading rooms in Manchester, his motive being to
stimulate interest in the local distribution o' infections sickness. Each
map bears indication, as nearly as possible in its appropriate position,
of every infect'ous attack reported in the course of the previous week,
different colonred spots representing the several nf t;fiable diseases.
Dr. Germain See communicated to the Academy of Medicine in
Paris, at its la*t sitting, a paper stating that he had reason to be satis-
fied with the results obtained by his new method of treating tnbtr-
culo is. By this method the patient if thut up in an air-tight cabm,
into which air, saturated with euoalvptol and creosote, is forced, th-
patient remaining in the box for four hours daily for several days.
Although not a cure for the disease, this method ?ecms to afford very
great temporary relief, and to check the progress of the malady.
Apbil 25, 1891.
THE HOSPITAL
The Student of Medicine.
[All communications for this Supplement should be addressed to The Editob of The Hospital, at 140, Strand, with the words, " Students*
Oolumn," written in the left hand top corner of the envelope.]
THE STUDENTS' BOOKSHELF.
"The St. Thomas's Hospital Gazette."*
St. Thomas's Hospital have just started a magazine of their
?wn, and have thus added to the few hospitals which are at the
present time publishing periodicals. The first number of this
enterprise which lies before us is, on the whole, very well got up,
and contains a good deal of subject matter. It starts with a
leader, entitled " Ourselves," explaining the reason d'etre and
the programme for the future. The main object of the paper is
to provide some medium of intercommunication between past
aad present students of the Hospital and Medical School. A
Publication Committee was started, and guaranteed among them-
selves a sufficient fund to avoid applying for pecuniary aid in any
'(Uarter. The artistic cover for the paper ha3 been designed by
Miss Andre. The paper is to contain reports of interesting cases
from the wards, articles useful for the senior students, also any
original work done in the Hospital or School or any new methods
of research. So also the proceedings of the Medical and Physical
Society, with abstracts of the papers read, which reports are to be
supplied by the writers of the papers themselves. So also the
doings of the various Athletic Clubs and other Students' Societies
yill be reported, with descriptions of the hospital matches.
Among the articles which follow, there is one on "Some Notes
on a Case of Probable Aortic Aneurism, under Dr. Ord," written
by Mr. W. W. Stabb; and then an article entitled " Sixty Years
Since," by Mr. F. Le Gros Clark, which contains a most interest-
^g account of the Hospital when the writer entered it sixty
years ago, giving an excellent description of the build-
111 gs at that period, and of many of the famous men
connected with it. This historical sketch will be read with
interest by all past and present Thomas's men. A full
description of the smoking concert and of the general
Qieeting of the Medical and Physical Society, and then the foot-
ball news follow, and the first number finishes with a page of
general hospital news, the results of the recent examinations, and
* "The St. Thomas's Hospital Gasette." Printed by William Olowes
ana Sons (Limited), Stamford Street and Charing Gross, London.
the last page of the journal is devoted to a concise table of tho
fixtures for the mouth. Examples of local enterprise such a&
starting and maintaining periodical literature by our leading
hospitals is to be commended, as it is a great means of bringing
the members of the staff into more intimate relation with the
students. So also it keeps up the interest of men who have long
since left their alma-mater for fields of active work, and who,
unless such a means as this exists, have little or no opportunity of
keeping in touch with their Hospital or of knowing of the sort
of work their centre of medical education is carrying on at the
present day, or, in fact, in some cases as to its existence at all.
We thus most strongly recommend all past and present Thomas's
men to take in their " Hospital Gazette,"and to help maintain the
high standard of scientific and literary excellence which the
editors have started by contributing articles and topics of medical
interest from the various centres of the world in which they are
now placed. We feel certain that the day will come when all the
leading schools will have their own magazines, and we shall be
glad to welcome the individual appearance of periodicals of this
nature.
APPOIlTTBZEirTS.
At St. Thomas's Hospital, Horace George Turney, M.A., M.B.r
M.S. Oxon, M.R.C.P. Lond., F.R.C.S., has been appointed
resident assistant physician. At the West London Hospital,
L. A. Bidwell, F.R.C.S., has been appointed assistant surgeon.
At St. Mary's Hospital, ?T. E.Lane, F.R.C.S., has been appointed
surgeon in charge of out-patients. At the Royal Hospital for
Children and Women, Waterloo Bridge Road, H. B. Osburn,
M.R.C.S., L.R.C.P., has been appointed anajthetist and registrar.
At the London Hospital, W. Howard Sturge, M.R.C.S., L.R.C.P.,
has been appointed house surgeon. At the Chelsea Hospital for
Women, G. Drummond Robinson, M.B., B.S. Lond., M.R.C.S.,
L.R.C.P., has been appointed resident medical officer. At the
North Staffordshire Infirmary and Eye Hospital, Stoke-upon-
Trent, Michael H. Ashwell, M.R.C.S., L.S.A., has been appointed
consulting physician, and Hubert Nicholls, M.B. Camb.r
M.R.C.S., assistant physician. At the Spalding Workhouse,.
THE HOSPITAL.
April 25, 1891.
THE STUDENT OP MEDICINE?(continued).
G. L. Barritt, L.R.C.P., M.R.C.S., ha8 been appointed medical
officer. At the Royal Southern Hospital, Liverpool, William
Andrew Betts, M.B., C.M. Edin., has been appointed junior
house surgeon. At the Carmarthen Infirmary, E. P. "Davies,
M.R.C.S., has been appointed consulting surgeon. At the Bethlem
Hospital, Howard Distin, M.R.C.S., L.R.C.P., has been appointed
resident clinical assistant. At the Adelaide Hospital, South
Australia, A. A. Lendon, M.D. Lond., M.R.C.S., has been
appointed honorary physician, and Harry Swift, M.B.,, M.D.
Camb., M.R.C.S., honorary assistant physician. At the Belfast
Royal Hospital, J. S. Morrow, B.A., M.B., has been appointed
house physician. At the Hanwell Asylum, R. L. S. Nutall,
M.R.C.S., L.R.C.P., has been appointed fifth assistant medical
officer. At the South Devon and East Cornwall Hospital, Ply-
mouth, A. W. F. Savres, M.R.C.S., L.R.C.P., has been appointed
assistant house surgeon. At the York Lunatic Asylum, Edward
Milliken Godlie, M.B., M.Ch. Edin., has been appointed assistant
medical officer. At the St. Marylebone General Dispensary,
William J. Radford, M.R.C.S., L.R.C.P., has been appointed
resident medical officer. At the Workhouse of the West Ham
Union, Ernest Vallance, L.R.C.P., M.R.C.S., has been appointed
assistant resident medical officer.
VACANCIES.
The following vacancies are announced this week, the amount of
salary in each case being given after the name of the institution :
Resident Medical Officers.
County Borough of Bradford, Fever Hospital, Resident Medical Superin-
tendent?Salary ?200, with Board, &c.
East London Hospital for Children, Shad well, B., House Physician?
Board, &c.
Glamorganshire and Monmouthshire Infirmary, Cardiff, Assistant
House Surgeon?Board, &e.
Guest Hospital, Dudley, Resident Medical Officer?Salary ?100, with
Board, &c.
Loughborough and District General Hospital and Dispensary, Resident
House Surgeon?Salary ?70, with Board, &c.
Manchester Hospital for Consumption and Diseases of the Throat, Resi-
dent Medical Officer at Bowden, Cheshire?Salary ?60, with
Board, &c.
New Hospital for Women, Euston Road, N.W., Female Resident Medical
Officer.
Noble's Isle of Man General Hospital and Dispensary, Douglas, Isle of
Man, Resident House Surgeon?Salary ?90.
North-West London Hospital, Kentish Town Road, Resident Medical
Officer, and Assistant Resident Medical Officer.
Nottingham Borough Asylum, Mapperley Hill, Resident Clinical Assis-
tant?Board, &c.
Sussex County Hospital, Brighton, House Physician and House Sur-
geon?Salaries ?80, with Board, &c.
Wallesay Dispensary, House Surg eon?Salary ?110.
We3t Bromwich District Hospital, Hemel Hempstead, House Surgeon
?Salary ?100, with Board, &c.
Wolverhampton Eye Infirmary. Resident Assistant?Board, &c.
York Dispensary, Resident Medical Officer?Salary ?130.
N"on-Resident Medical Officers.
Charing Cross Hospital Medical Sohool, Demonstrator of Physiology?
Honorarium ?100.
St. George's and St. James'3 Dispensary, King Street, Regent Street?
Third Honorary Physician.
Western Skin Hospital, Great Portland Street, W., Honorary Physician.
SCHOLARSHIPS AITS FRIZES.
University of Aberdeen?Winter Session, 1890-91.
Fife Jamieson Memorial Gold Medal in Anatomy, awarded to James
Gillespie, Aberdeen, and John T. West, Dinnet. Keith Gold Medal for
Systematic and Clinical Surgery, awarded to Alexander Grant. Shepherd
Memorial Gold Medal for Systematic and Practical Surgery, awarded to
James Rust, M. A. Medals in Clinical Surgery, Descriptive Case Taking,
awarded to William Trethowan and Alexander Grant, equal.
PRESENTATION.
Mr. E. Wilmer Phillips, L.R.C.P., M.R.C.S., having resigned the office
of House Surgeon of the Windsor Royal Infirmary, which post he has held
for five years, has been presented with a valuable gold watch, subscribed
for chiefly by the staff of the Institution, together with some of the
managers and several of the patients.
PASS LISTS.
University of Durham.
FACULTY OP MEDICINE.
Examinations for Degrees in Medicine and Surgery, at the
College op Medicine, Newcastle-upon-Tyne, April, 1891.
First Examination for the Degree of Bachelor in Medicine.
The following candidates have satisfied the examiners :?
Elementary Anatomy and Physiology, Chemistry with Chemical
Physics, and Botany with Medical Botany.?Thomas Cook Barkas,
College of Medicine, Newcastle-upon-Tyne; Edward James Brewis, Col-
April 25,1891. THE HOSPITAL,
THE STUDENT OF MEDICINE?(continued).
lege of Medicine, Newcastle-upon-Tyne 5 James William Gibson, College
-of Medicine, Newcastle-upon-Tyne; Sidney Herbert Hawley, Queen's
College, Birmingham; Christopher Julius Jeaffreson, College of Medi-
cine, Newcastle-upon-Tyne; George Edwin Pearcey, College of Medicine,
Newcastle-upon-Tyne ; George William Pickering, Queen's College, Bir-
mingham ; William Hunter Richards, College of Medicine, Newcastle-
upon-Tyne; William Smith, College of Medicine, Newcastte-upon-Tyne ;
and William Edwyn Falkingbridge Tinley, College of Medicine, New-
castle-upon -Tt ne.
Elementary Anatomy and Physiology.?Richard Henry Bellwood,
College of Medicine, Newcastle-upon-Tyne; Harry Bond, Colletre of
Medicine, Newcastle-upon-Tyne; William Ewart Gibbs, College of Medi-
cine, Newcastle-upon-Tyne; Harry b arte Gourley. Edinburgh School of
Medicine; Charles Hanks, College of Medicine, Newcastle-upon-Tyne;
Alfred Phillips Lloyd, College of Medicine, Newcastle-upon-Tyne; Robert
Alexander Morris, College of Medicine, Newcastle-upon-Tyne; Edward
Tonge, College of Medicine, Newcastle-upon-Tyne; and Charles Robinson
Wood, B A., College of Medicine, Newcastle-upon-Tyne.
Chemistry with Ch- mical Physics, and Botany with Medical
Botany.?Percy Luke Armstrong, College of Medicine. Newcastle-upon-
Tyne ; Percy Robert Ash, Yorkshire College, Leeds ; William Gladstone
Cook, College of Medicine, Newcastle-upon-Tyne; William Wilkie
Deans, Yorkshire College, Leeds , William Georgo Pell, College of Medi-
cine, Newcastle-upon-Tyne; Patrick Aloysius Joseph Vincent Porde,
8t. George's Hospital; Ernest Rowland Pothergill, Guy's Hospital;
Neville Claude Gwynn, Bristol Medical School; Henry Hartigan,
Medical School, Catholic University, Dublin; Thomas Hartigan, Medi-
cilSchool, Catholic University, Dublin; Edward Augustus Lermitte,
College of Medicine, Newcastle-upon-Tyne; Walter Frederic Miller,
Guy's Hospital; William Harland Peake, Guy's Hospital; Thomas
James Selby, Edinburgh School of Medicine; Edward Ernest Wood-
house, College of Medicine, Newcastle-upon-Tyne; Edwin Percy Wrinch,
St. Thomas's Hospital.
Chemistry with Chemical Physics.?Percy Coleman, M.R.C.S.,
L.R.C.P.,Lond., St. Thomas's Hospital; Louis George Charles Yintras,
M.R.C.S., L.R.C.P., B.Sc.Paris, St. Mary's Hospital.
Examining- Board in England by the Royal Colleges of
Physicians and Surgeons.
The following gentlemen passed the Second Examination of the Board
at a meeting of the Examiners on the 9th inst.:?
Anatomy and Physiology.?W. E. Leetham Horner, Joseph E.
Waite, T. Villiers Crosby, and G. H. Oawley Way, of University College;
John W. Haines, Alexander C. Gurney, J. Burnell Great-Rex, G. Ray-
mond Fox, Courtney H. Drake, and J. Littleton Dalby, of St. Bartholo-
mew's Hospital; Edward Bromet, Alfred E. Russell, Sidney W. F.
Richardson, and Henry W. Harding, of St. Thomas's Hospital; Charles
E- Cock and William T. White, of Charing Cross Hospital; 0. Kemble
James, of Middlesex Hospital; Nathaniel D. S. Towne and 0. Poulett
Harris, of London Hospital; W. Oharrragton Wood and Mark A. Salt-
marsh, of St. Mary's Hospital; F. Kenneth Wilson, of Westminster
Hospital; and Oonrtney R. Oolley, of Guy's Hospital.
Anatomy only.?Dan Davies, of London Hospital; Leonard J. Miskin,
of St. Mary's Hospital; Oharles H. Nicholson, of Middlesex Hospital;
H. Gallway Larnder, of St. Mary's Hospital; and Barnet Saul, of
Oharing Cross Hospital.
Physiology only.?Hubert Williams, of Middlesex Hospital; A.
Walter Austen and William H. Ohute, of Westminster Hospital; and
Ernest G. Annis, of Guy's Hospital.
Passed on the 10th inst.
Anatomy and Physiology.?L. E. V. Every. Clayton; of Guy s Hos-
pital; Albert W. Riley and William H. Thomas, of London Hospital;
Frederic Cecil Robinson, Walter H. Pollard, E. LawsonPawlett, Harold
B. Meakin, and Thomas Hampton, of St. Bartholomew's Hospital; W.
West Linnington, Antbony A. Martin, Robert Corfe, and Colin D.
Lindsey, of St. Mary's Hospital; John Harper and Arthur Dobson, of
St. Thomas's Hospital; F. Nesfield Cookson, of Middlesex Hospital;
Charles E. Wallis and Frederick C. Spawson, of King's College; H.
Matthew Dowler and 0. Dudley Garrett, of Westminster Hospital; and
H. R. Belcher Hickman, of St. George's Hospital.
Anatomy only.?Hobart J. W. Barlee and Francis H. Bailey-King, of
Middlesex Hospital; Philip G. Williams, of London Hospital; and Arnold
Stockton, of King's College.
Physiology only.?Arthur Barnes, of Charing Cross Hospital;
Alexander M. Watta, of University College; Frederick A Cooke, of St.
Mary's Hospital; William E. Lee, of St. Bartholomew's Hospital; and
Archibald J. Campbell, of Sc. Thomas's Hospital.
Passed on the 13th inst.:?
Anatomy and Physiology.?J. Macpherson Stewart and T. Bernard
Stedman, of University College ; Oharles G. Cory and Sydney Cornish,
of St. Bartholomew's Hospital; W. Archibald Propert, of St. George's
Hospital; Wilfrid Fleming, of Westminster Hospital; Daniel E. Evans,
H. Gwynne Lawrence, Edgar J. Morgan, and G. Bushman Riddick, of
St. Mary's Hospital; J. E. Gilbert Rogers, of London Hospital, Percy
J. Probyn and T. Elgee Cundy, of Charing-cross Hospital; Charles H.
Watson and W. Poce Seed, of Middlesex Hospital; R. Hilton Fagge and
G. Leslie Eastes, of Guy's Hospital.
Anatomy only.?Leonard Harman, of St. Thomas's Hospital; A;
Condell Strand,of Middlesex Hospital; Herbert 0. Wimble, of St. Bar-
tholomew's Hospital and Mr. Cooke's School of Anatomy and Physio-
logy ; and J. West Summerhays, of St. Mary's Hospital.
Physiology only.?T. H. Pryce Morris, of Middlesex Hospital;
William R. Larbelestier and H. Walker Whitley, of Charing-cross Hos-
pital; H. Naunton Robson, of St. Thomas's Hospital; and Oharles H.
Wilmer, of St. Bartholomew's Hospital and Mr. Cooke's School of
Anatomy and Physiology.
THE HOSPITAL. April 25,1891.
THE STUDENT OP MEDICINE-[continued).
Passed on the 14th inst..
Anatomy and Physiology.?William Gardner and John Garrett, of
St. Mary's Hospital; Hugh M. Rigby, Victor J. Batteson, and Harry
E. B. Porter, of London Hospital; Thomas W. W. Burgess, Percy 0.
Barford, Evelyn G. B. Adams, Percy E, Adams, Frederick W. Robert-
son, and Percy E. Turner, of St. Bartholomew's Hospital; Walter F.
Henley, of Charing Gross Hospital; John F. Sarjeant, William Way,
Wilfred Hudleston, and John Kekwick, of Middlesex Hospital; Eli is
F. Harwood, Arthur P. Nuttall, John P. Kitson, and Thomas R.
Llewellyn, of University College; Frederick S. Penny, of King's Col-
lege; George A. Oiarkson, of St. George's Hospital; and Arthur F. W.
King, of St. Thomas's Hospital.
Anatomy only.?Harold J. K. Bamfield, John H. Campbell, and
John B. Yelf, of St. Mary's Hospital; Henry A. Andrews, of St. Bar-
tholomew's Hospital; Edgar J. Rowbotham and Hubert A.Madge, of
Charing Cross Hospital; and Walter L. Stuart, of Gay's Hospital.
Physiology only.?Moses J. Reubens, of Grant Medical College,
Bombay.
Passed on the 15th inst :?
Anatomy and Physiology.?Reginald N. Weekes, H. T. M. Whitling,
0. Mackenzie Hew^r, and Robert H. Wilkin, of St. Bartholomew's
Hospital; 0. Beauchamp Hall, of St. Mary's Hospital ; Moyle Breton,
of St. George's Hospital; F. J. Goodwin and Edmund B. Jones, of
Charing Cross Hospital; W.David Jones, Adam Cowes, and J. Lloyd
Davis, of University Oolleee; Charles H. Dickens and P. Olennell Fen-
wick. of St. Thomas's Hospital; Henry W. Collier, Guy E. Manning,
and Harold P. Barker, of Guy's Hospital; Martin W. Loy, of London
Hospital; Mark Farrant, of Westminster Hospital; and Ernest Playfair,
of King's College.
Anatomy I only.?W. Berkeley Murray and Richard S. Olver, of King's
College; Alfred J. Grant, of St. Thomas's Hospital; and William
Wyllys, of St. Bartholomew's Hospital.
Physiology only.?T. Dobson Bell, of University College; Arthur
J. Mathison, of Guy's Hospital; and George R. Harcourt, of St.
Thomas's Hospital.
St. Andrews, April 16th.
At the annual graduation ceremonial the following gentlemen, having
passed the required examinations, had the degree of Doctor of Medicine
conferred upon them: James Wallace Anderson, Glasgow; Richard
Henry Barker, Hungerford; Andrew Graham Currie. Francis Hollins-
head, Selly-Oak; John Hay Honeyman, Auckland, N.Z.; John Fletcher
Home, Barnsley; Lawrence Storrar Mackenzie, Bradford; John Nairn,
Glasgow; Frederick La Coque Thome, Leamington; and Albert
Wheeler, London.
University of Glasgow.
The following gentlemen have passed the First Professional Examina-
tion for the degrees of Bachelor of Medicine (S?.B.) and Master in
Surgery (O.M.): John Ewin Adam, Andrew Phillips Aitken. John Allan.
John Gibson Anderson, John Bain, D ivid Blair, M.A., James Boyle,
Walter Buchanan, Edward Napier Burnett, Archibald Campbell,
Thomas R. A. A. G. Crawford, Thomas Orossan, James Donaldson,
Duncan Drummond, William Moyes Duff, James Findlay, William.
David Findlay, Robert Scott Frew, Robert Howieson Gemmell, Ernest
William Graham, Thos. Holmes, William Humphreys Jamison, Edgar
Milwynne Jenkins, John Gonnan Lander, Jotin Herbert Liwson, John
Joseph Lowell, James Wm. van Millingen, Alan Muir, Wm. Edgar
Macharg, Ohas. M'Kiy, John M'Kenna, Jas. Campbell M'Neillie, Alfred
George Newell, Peter Patton Petrie, M.A., Wm. Salmond, Joseph
Scjtt, John Selkirk, M.A., Henry Beveridge Smith, Thos. David Smith,
James Thomson, M.A., and Rjbert Nicol White.
The following gentlemen have passed the Second Professional Exami-
nation : Walter Isaac Buchanan, Archibald Prsn'ice Campbell, David
James MacGoun Campbell, William Campbell, John Joseph Garrathers,
David Charters, Thomas Colvin, William Copland, William James
Couper, Robinson Ruddock Cojle, James Conway D,ivies, John Stanley
Davies, M.A., George Edgar, James Donald Finlay, Alfred Forrest,
M.A., Henry Niven Gardiner, Alfred Alex. Gumming Grant, David
Fraser Harris, B.Sc., William Munn Hunter, James Jack, Henry
Edward Jones, Alexander Kelso, David Millar King, William Anderson
Kirkwood, James Lang, Robert Langmnir, M.A., Robert M'Farlane
Marshall, Thomas Burns Marshall, Jamea Binnie Millar, David Ram-
fay Miller, Edward James Morris, John Morrison, Wm. M'NivenMuat,
Wm. Muir (Paisley), Colin Campbell M'Call, Alex. Hutchison M'Cracken,
James Morton Mac Lauchlan, John Alfred Naismith, George Nicoll,
John Paxton, James Pet'igrew, Euseno Louis Polonais, Archibald
Robertson, M.A., Hector Monteith Robertson, Francis Small. Nathaniel
Stevenson, M.A., Robert Tennent, James Thompson, Alfred Webster,
John Cameron Young.
The following gentlemen have passed the Third Professional Exami-
nation, including, in the cases marked with asterisks, the subject of
Pathology James Aitken, William Limont Brown, Adam Guthrie
Burrell, 'Alexander Baillie Craig, John Alexander Creighton, John
David Davies, Donald Stewart Dewar, Robinson Simpson Dickson, Thos.
Divine, *Neil Downie, *Andrew Findlay, Robert Fureyth, *David
Frame, M.A., *David Wyllie Girvan, *John Green, John Atkinson Har-
rison,. James Hunter, Hugh Kerr, M.A., David Clark Laird,'Peter
Alexander Laird, James Lamont, John Lindsay, M.A., Jas. Buchan
Littlejohn, Samuel T. M'Clintock Lowry, 'Robert Mortimer Malcolm.
?William Cochrane Murray, Alex. Christie M'Arthnr, Donald Douglas
Macdonald, 'Donald Neil Macfarlane, "John M'Gregor, 'Charles Hugh
M'llwraith, M.A., Alexander John M'Kechnie, John Pearson, Henry
Joseph Rankin, 'David Smith, Peter Maclem Smyth, 'Maurice Tansred
Stack, 'William Steel,'William Caldwell Steele, John Stewart, David
Robert Thomson Strong, 'George Mervyn Sydenham, 'James Allan
Wilson, Charles Frederick Wylie.

				

